# Efficacy of Antivascular Ultrasound (AVUS) in Hepatocellular Carcinoma (HCC)

**DOI:** 10.3390/cancers16223756

**Published:** 2024-11-07

**Authors:** Moein Moradpour, Zhuorui Li, Omar Al-Daoud, Fereshteh Yazdanpanah, Aashish Cheruvu, Chandra Sehgal, Stephen Hunt

**Affiliations:** Department of Radiology, Hospital of the University of Pennsylvania, 3400 Spruce St., Philadelphia, PA 19104, USA

**Keywords:** antivascular ultrasound (AVUS), ultrasound, minimally invasive, locoregional therapy, interventional radiology, interventional oncology, liver cancer, hepatocellular carcinoma (HCC)

## Abstract

Background: Hepatocellular carcinoma (HCC) is a prevalent type of primary liver cancer and one of the leading causes of cancer-related mortality worldwide. Antivascular Ultrasound (AVUS) is a novel therapy approach that utilizes the mechanical and thermal interactions between ultrasound and microbubbles to disrupt tumor vasculature or potentiate effects of chemotherapy or radiation therapy in a dose-dependent fashion. In this review, we aim to illustrate the mechanisms of AVUS, focusing on the preclinical and clinical evidence of AVUS applications in HCC. Methods: Peer-reviewed publications pertaining to the use of AVUS in HCC were collected and analyzed. Results: 12 preclinical studies and 1 clinical trial were analyzed. At lower energy levels, AVUS can enhance tumor perfusion, facilitating the delivery of chemotherapy agents and resulting in improved therapeutic outcomes. Conversely, at higher energy levels, AVUS can disrupt tumor perfusion, leading to ischemic damage of the tumors. Combining AVUS with other therapeutic approaches, such as chemotherapy, radiation therapy, and transarterial radioembolization (TARE), can synergistically enhance therapeutic outcomes. Conclusions: AVUS is a promising novel treatment modality for HCC. Current evidence suggests that AVUS exhibits a dose-dependent nature, making it a versatile approach that can be effectively combined with other therapeutic regimens. Further clinical studies and long-term follow-ups are needed to establish the optimal clinical protocol and safety profile of AVUS.

## Simple Summary

Antivascular Ultrasound (AVUS) is a novel therapy that combines low-intensity ultrasound and contrast microbubbles to disrupt tumor vasculature, providing a potential treatment for hepatocellular carcinoma (HCC). In a dose-dependent fashion, AVUS can leverage the mechanical and thermal effects of ultrasound-induced microbubble oscillations to disrupt the vasculature of tumors and induce ischemic damage to tumor cells. Conversely, lower energy application can enhance tumor perfusion, predisposing tumors to better chemotherapy response. Preclinical and clinical studies have demonstrated its efficacy is dose-dependent. In combination with traditional treatments such as chemotherapy and transarterial radioembolization (TARE), AVUS has shown synergistic effects, significantly improving therapeutic outcomes. While AVUS shows promise as a minimally invasive and cost-effective treatment for HCC, further clinical trials are required to establish an optimum therapeutic protocol and safety profile. As research progresses, AVUS may emerge as a valuable treatment for hepatocellular carcinoma, offering a noninvasive option for patients.

## 1. Introduction

Hepatocellular carcinoma (HCC) is the most prevalent primary liver cancer, ranking fifth globally among cancers affecting men and seventh among women. It is the second most frequent cause of cancer-related deaths worldwide. Most HCC cases are linked to chronic liver dysfunction arising from hepatitis B virus (HBV), hepatitis C virus (HCV), alcoholic liver disease (ALD), and non-alcoholic fatty liver disease (NAFLD) [1,2]. The curative treatment options for HCC include early surgical removal or liver transplant for eligible patients. Unfortunately, less than 20% of patients are candidates for definitive surgical resection at the time of presentation. Percutaneous ablation provides a minimally invasive curative therapy for those patients presenting with a small number of tumors within specific size and location criteria. Other image-guided locoregional therapies (LRTs) such as transarterial embolization (TAE), transarterial chemoembolization (TACE), transarterial radioembolization (TARE), and transarterial immunoembolization (TAI) offer additional treatment options for patients who are not candidates for definitive surgical resection, transplantation, or ablation. Unfortunately, the majority of patients experience recurrence with these therapies, and retreatment is often limited by underlying liver dysfunction or vascular changes brought about by the prior therapy. In addition, HCC often arises in the context of cirrhosis, which represents a therapeutic challenge due to limited hepatic reserve. Antivascular ultrasound (AVUS) presents a promising alternative noninvasive locoregional therapy that targets the tumor’s blood vessels and can be used alone or in combination with other locoregional therapies [3]. It may provide a safer alternative to existing therapies in the setting of cirrhosis. This review provides an overview of this technology and its application to HCC treatment.

## 2. Basic Principles

**Figure 1:**
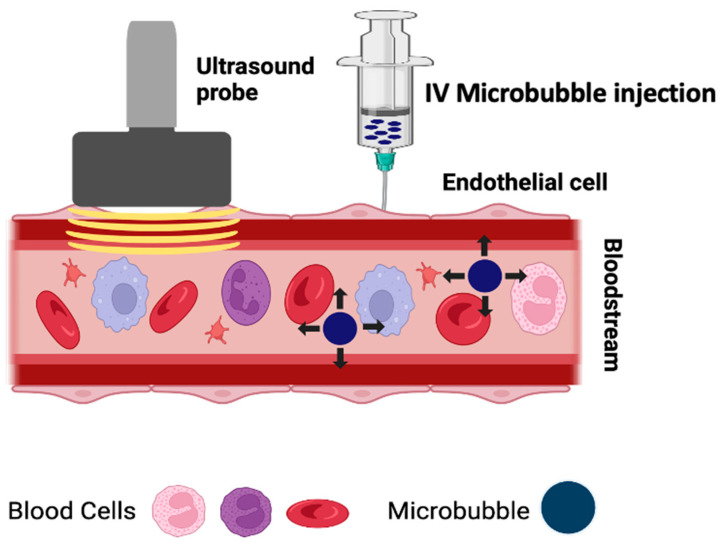
AVUS is a combination of low-intensity ultrasound and intravascular microbubbles. Microbubbles driven by ultrasound oscillate, collapse, and generate heat and mechanical forces to disrupt the tumor vasculature.

**Figure 2:**
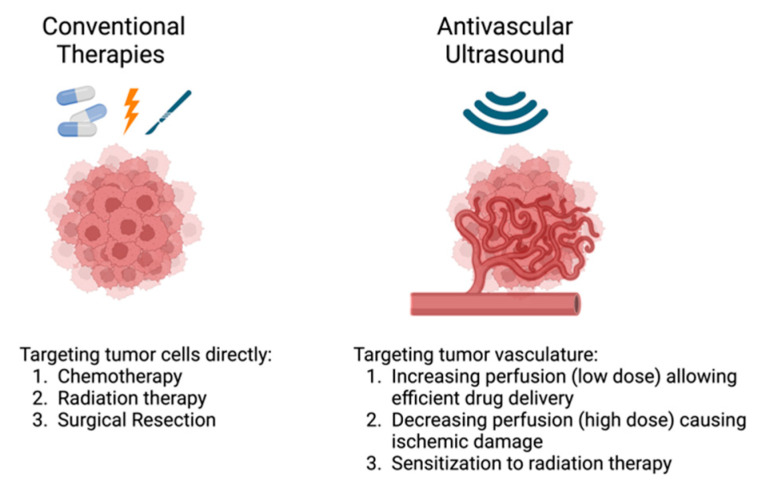
Traditional cancer therapies target the tumor cells directly, while AVUS targets the tumor vasculature, leading to dose-dependent responses.

AVUS combines low-intensity ultrasound with ultrasound contrast microbubbles injected into the vasculature (Figure 1). The ultrasound energy induces the oscillation or collapse of these microbubbles, creating focal hot spots, shear stress, cavitation, and microstreaming. In essence, with the correct pairing of ultrasound frequency and microbubble size, the microbubbles function as energy transducers to translate the ultrasound energy into heat and mechanical forces. Depending upon the intensity of the treatment, AVUS can temporarily modulate the tumor vasculature to make it more porous for drug delivery or permanently impair the tumor vasculature, causing vascular disruption and thrombosis (Figure 1) [4]. This is analogous to reversible electroporation, which is used to temporarily open pores in the cellular membrane for molecular delivery, versus irreversible electroporation, which permanently damages the cell membrane, resulting in cellular death. Rather than targeting the tumor cells directly, AVUS targets the vascular endothelial cells, causing tumor cell death due to insufficient oxygen and nutrients reaching the affected area (Figure 2) [3]. Because of the low-intensity nature of the ultrasound to induce therapeutic effects, AVUS offers a theragnostic platform in which both treatment and monitoring of the treatment effects can be conducted using the same ultrasound probe by simply adjusting the ultrasound’s operational mode [5]. In AVUS therapy, Monitoring the effects of tumor perfusion can be achieved using contrast-enhanced ultrasound (CEUS), where the nonlinear contrast mode detects microbubble-induced harmonic frequencies for high contrast-to-tissue perfusion imaging, while the power Doppler mode may capture low-velocity blood flow in small vessels [5]. This allows for real-time assessment of vascular responses using parameters such as peak enhancement (PE), perfusion index (PI), time to peak (TTP), and Doppler area and intensity, enabling adjustment of the treatment parameters as needed during treatment [5,6].

In conclusion, AVUS can either temporarily make tumor blood vessels more porous for drug delivery or permanently damage them, leading to tumor cell death due to a lack of oxygen and nutrients. The theragnostic AVUS system, combining imaging and therapy, can perform treatment monitoring and dose adjustment in real-time without requiring expensive cross-sectional imaging like CT, MR, or angiography. Such a system is well suited for use in resource-limited settings.

## 3. Targeting the Tumor Vasculature

In contrast to regular blood vessels, tumor vasculature exhibits an irregular course, loose junctions between endothelial cells in the vessel wall and abnormal endothelial basement membranes and often lacks proper structural elements like pericytes. While many tumors such as HCC are hypervascular, this vasculature is often defective, resulting in increased permeability and delayed and incomplete vascular maturation. These characteristics make the vessels within the tumor more fragile compared to normal blood vessels and more susceptible to vascular disruption [7]. The disorganized and tortuous nature of the tumor vasculature also results in a slower blood flow and longer transit time through the tumor. In the setting of AVUS, this allows each microbubble to stay in contact with the tumor vasculature and ultrasound treatment field for a longer duration compared to healthy blood vessels in normal tissue [4]. This allows for a therapeutic window in which the impact of AVUS is primarily confined to the tumor’s blood vessels, exerting minimal influence on healthy surrounding tissue vasculature [8].

AVUS operates similarly to pharmacologic vascular disrupting agents (VDAs) like combretastatin [4]. VDAs act as inhibitors of angiogenesis, disrupting the tumor’s vasculature, causing ischemia and tumor cell death [4]. However, VDAs often result only in the central parts of the tumor undergoing necrosis, leaving viable cells at the margin that can regenerate into a more aggressive tumor. In addition, VDAs affect neovascularization in normal tissues, causing off-target effects that result in hemorrhage and other vascular complications. AVUS disrupts the tumor vasculature within a treatment field, including both the center and peripheral aspects of the tumor; however, its effects are limited to the treatment area, which results in a more complete treatment with a lower risk of off-target effects. By inducing cellular stress in tumors and free-radical formation during cavitation, AVUS has also been used to sensitize cancerous tissue to radiation therapy, similar to the radiation-sensitizing effects of VDAs [8].

In practice, pre-treatment diagnostic CEUS is performed to confirm hyperenhancement with microbubble administration. Mounting the therapeutic probe with a diagnostic probe, along with the previously described extended contact time of a microbubble with tumor vasculature in contrast with normal tissue, allows for accurate targeting of the treatment field [5].

## 4. Comparison to Focused Ultrasound

**Figure 3:**
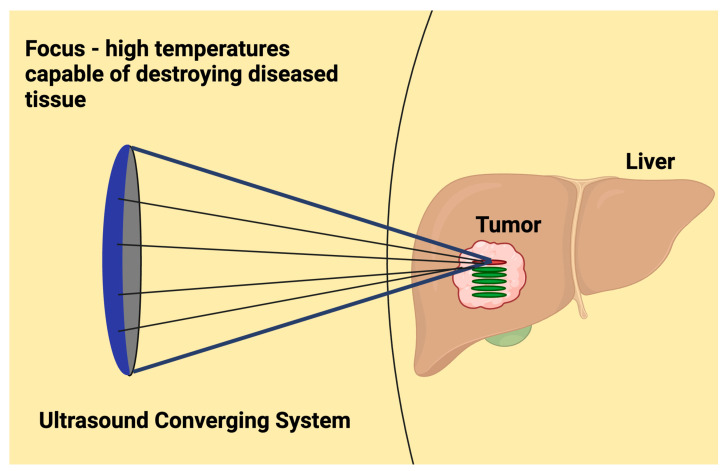
HIFU uses very high-intensity focused ultrasound energy to raise the temperature of the targeted area, causing direct thermal ablation of tumor tissues.

Focused ultrasound surgery (FUS) uses specialized focused ultrasound arrays to generate a high-intensity focused ultrasound (HIFU) to destroy targeted tumor tissue directly, rather than targeting the tumor vasculature [9]. HIFU transducers generate an intensity exceeding 1000 W/cm^2^ within a small tissue volume, rapidly inducing heating and cavitation effects. HIFU elevates the target tissue to high temperatures (65 to 100 degrees Celsius), resulting in thermal ablation (Figure 3) [10]. On the other hand, AVUS therapy operates at intensities below 5.0 W/cm^2^, which allows the therapy to be generated using standard diagnostic ultrasound probe technology, inducing weaker thermal effects with decreased risk of damage to normal tissue [11].

## 5. Mechanism

### 5.1. Microbubbles

Microbubbles are employed as contrast agents in CEUS [12]. They can be used in monitoring the response to anticancer treatments that impact tumor blood flow, as they enhance the visibility of the tumor vasculature [4]. They typically comprise a lipid, polymer, or protein shell, encapsulating a gas core with diameters ranging from 1 to 8 μm [4]. Their size allows them to pass through the lumen of capillaries while being large enough to prevent leakage from vessels, thus remaining intravascular [4]. Microbubbles can be modified to carry chemotherapy or be linked with drug nanoparticles, liposomes, or ligands. They can also serve as protective carriers for antibodies, RNA, or DNA to shield the molecules from enzymatic degradation prior to their deposition in the tumor tissue.

**Figure 4:**
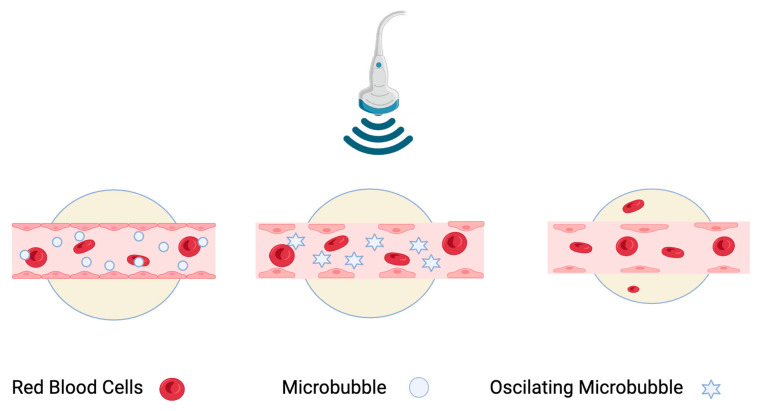
During AVUS, microbubbles function as cavitation nuclei experiencing compression, expansion, and collapse within the ultrasonic field. This leads to a series of mechanical effects that can increase vascular permeability or result in permanent vascular rupture and thrombosis.

In AVUS, microbubbles act as cavitation nuclei, undergoing compression, expansion, and collapse within the ultrasonic field (Figure 4) [13]. These oscillations cause heating and mechanical effects like microstreaming and liquid jets, enhancing interaction with the endothelium. Higher concentrations of microbubbles increase energy deposition and local heating, potentially improving therapeutic effects by bringing more bubbles close to the tumor’s endothelial cells.

Common commercially available microbubbles include Levovist^®^ (Bayer Schering, Berlin, Germany), Optison^®^ (GE Healthcare, Princeton, NJ, USA), SonoVue^®^ (Bracco Company, Milan, Italy), Definity^®^ (Lantheus, North Billerica, MA, USA), and Sonazoid^®^ (GE Healthcare, Princeton, NJ, USA). Specific to the application of AVUS in HCC, studies have most often chosen Definity^®^ (Lantheus, North Billerica, MA, USA) due to its wide availability, or SonoVue^®^ (Bracco Company, Milan, Italy), due to improved harmonic behavior at low power and minimal phagocytosis by Kupffer cells [14]. However, to our knowledge, no direct comparison of different microbubbles has been performed in the context of AVUS in HCC.

### 5.2. Ultrasound Parameters

The AVUS effects on tumor vasculature depend on the depth of the scanned area, the frequency of sound waves, the ultrasound intensity, the pulse length and duty cycles of the pulsed ultrasound, and the duration of the application. The adjustments of these parameters allow for controlled and targeted interaction with (and, if desired, destruction of) microbubbles within the tumor [15]. For example, the scanning area can be targeted toward the residual enhancing portions of the tumor, the duty cycle can be increased to allow a higher percentage of treatment time spent in continuous isolation, the ultrasound power can be increased to enhance bubble destruction if trying to deliver a payload, and the duration of treatment can be altered. A shorter isolation time decreases the effects on the microbubble-related activity. Similarly, a longer interval between sonication pulses allows for the preservation of intact microbubbles and enhances microbubble inflow into the tumor [7].

## 6. AVUS Dose Dependence

**Figure 5:**
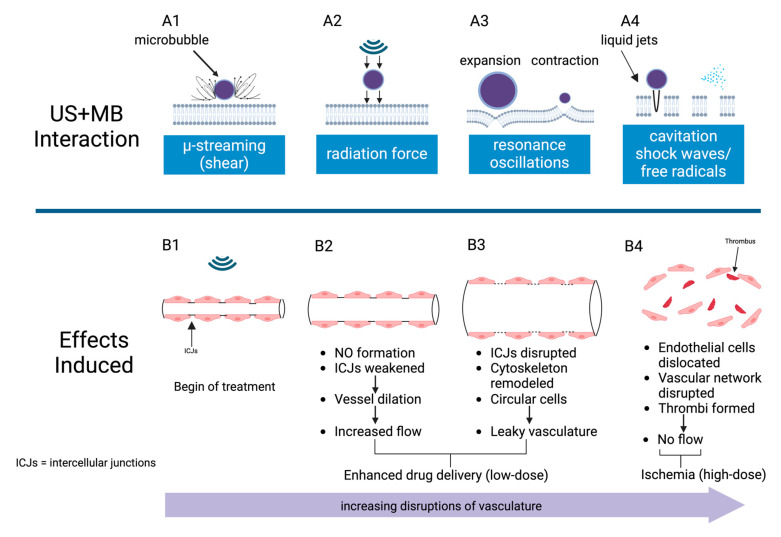
AVUS induces diverse mechanical forces on the vasculature, including microstreaming (**A1**), radiation force (**A2**), resonance oscillation (**A3**), and cavitation (**A4**). Prior to treatment, tumor vasculature has intact intercellular junctions (ICJs) (**B1**). At lower intensities, AVUS triggers the formation of nitrous oxide, leading to vascular dilation, increased blood flow, and increased permeability (**B2**,**B3**). These effects can be used to increase drug delivery. Higher-intensity AVUS permanently disrupts the vascular network, causing vascular rupture, thrombosis, and ischemic damage to tumor cells (**B4**).

If the therapeutic intent is ischemic cancer therapy, decreased blood flow is essential to induce ischemia. In this setting, the ultrasound parameters and microbubble dose can be optimized for maximal vascular disruption and thrombosis. At higher energies, this results in permanent injury to the vascular walls and triggers thrombotic effects in the tumor vessels and ischemia of the tumor tissue (Figure 5).

Alternatively, ultrasound treatments at lower doses can enhance tumor perfusion (Figure 5). Enhanced tumor perfusion is often required for optimal therapeutic drug delivery and the effectiveness of radiation therapy. Tumor perfusion and flow distribution within the tumor vasculature dictate the tumor’s exposure to chemotherapy. The heightened concentration of chemotherapy agents in the tumor tissue promotes tumor necrosis [12]. In radiation therapy, oxygen plays a critical role as it generates free radicals that effectively eliminate cancer cells. Diminished oxygen levels within a tumor can result in decreased efficacy of radiation therapy. Therefore, increasing tumor blood flow can enhance treatment effectiveness [16].

The interactions between microbubbles and endothelial cells are complex and influenced by various factors, including the conditions of ultrasound exposure and the concentration of microbubbles. By carefully controlling these parameters, it is possible to enhance specific interaction mechanisms selectively. This approach can lead to a variety of vascular bioeffects for HCC treatment, such as improved blood flow, increased vascular permeability, and even the induction of ischemia.

## 7. Preclinical Evidence for AVUS in HCC

**Figure 6:**
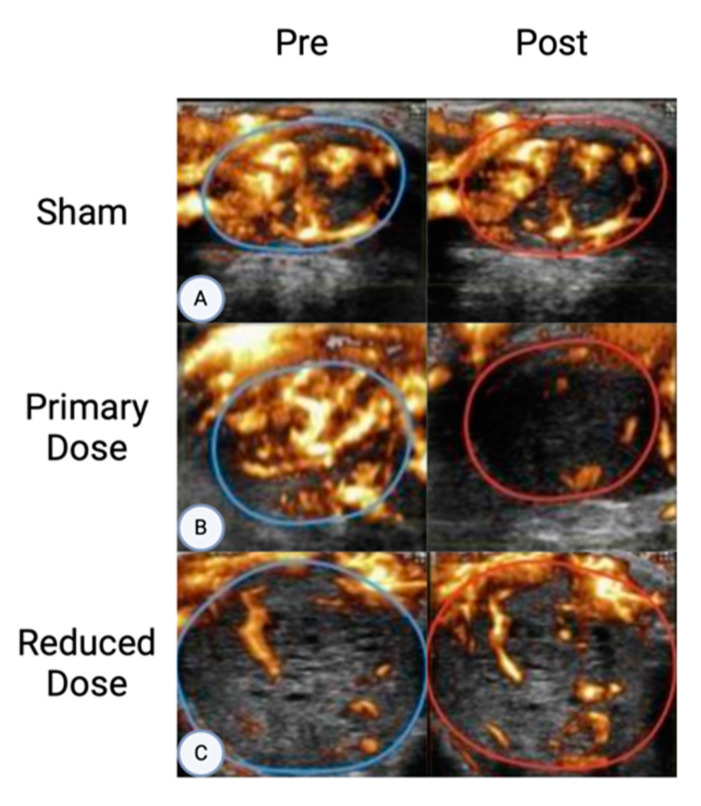
Power Doppler images pre- and post-AVUS adapted from D’Souza et al. show dose-dependent responses of AVUS within targeted areas (indicated by circle regions of interest) [5]. The sham treatments (**A**) show equivalent perfusion before and after therapy. After treatment of a primary dose (**B**), a significant reduction in tumor perfusion can be visualized. Reduced-dose therapy (**C**), while showing a variable response overall, showed an increased perfusion in the majority of cases.

Preclinical studies have supported two separate dose-dependent treatment effects (Figure 5). In a preclinical HCC study comparing low- and high-dose AVUS regimens, 19 rats received a standard AVUS treatment regimen (I_sata_ = 2-W/cm^2^, 3-MHz, 6 min of continuous insonation with 0.7 mL of microbubbles), while 11 rats were administered an alternate lower dose (I_sata_ = 1.0-W/cm^2^, 3-MHz, 3 min of continuous insonation, 0.1 mL microbubbles) [5]. Following the standard dose, the average decrease in perfusion between pre- and post-peak enhancement was measured at 37.9% ± 10.05% [5]. With the lower dose, there was an average increase in perfusion by 38.3% ± 26.2%, although not statistically significant [5]. This dose-dependent response to AVUS is showcased in Figure 6, where nonlinear contrast and power Doppler images clearly demonstrate reduced perfusion following the standard dose compared to sham treatment, while lower doses led to increased perfusion [5]. In a similar preclinical rat model of HCC, 22 rats with HCC were categorized into three groups: a low-dose AVUS group (*n* = 7) receiving treatment with I_sata_ of 1 W/cm^2^ for 3 min with 0.3 mL microbubbles, a high-dose AVS group (*n* = 12) exposed to treatment with I_sata_ of 2 W/cm^2^ for 6 min with 0.7 mL microbubbles, and a sham group (*n* = 3) that did not undergo AVUS. The peak enhancement and perfusion index decreased by 58.1% and 49.1%, respectively, after high-dose AVUS, while they increased by 47.8% and 20.3% following low-dose AVUS [16]. The reduction in both the peak enhancement and perfusion index after high-dose AVUS confirms its potential application in tumor devascularization. Conversely, the observed increase in perfusion parameters following low-dose AVUS indicates its potential for enhancing drug delivery or aiding in radiation therapy [8,16].

AVUS can also be repeated to maximize its effect. The timing between AVUS treatment sessions also influences the efficacy of the therapy. A variable proportion of tumor vessels may be nonfunctional and lack blood flow at certain times, so repetitive subsequent treatments that target vessels at different stages can surpass the limitations of a single treatment. A study involving 24 rats with HCC included three groups: single therapy (ST), 2-days subsequent (2DST), and 7-days subsequent (7DST) [3]. The 2DST group exhibited the most significant reduction in tumor perfusion, measured at 75.3%. The ST group experienced a decrease of 54.3%, while the 7DST group showed the smallest reduction in perfusion, measured at 45.3% [3]. The enhanced effects observed with subsequent treatments within a shorter interval could be attributed to the tumor’s increased susceptibility two days after the initial therapy. Changes within the tumor, such as necrosis and reduced vascularity, might render it less responsive to vascular targeting further out from initial treatment [3].

AVUS has a remarkable effect on tumor perfusion. In a preclinical rabbit model of HCC, AVUS displayed a 70% reduction in perfusion along with a substantial decrease in tumor growth rate compared with ultrasound or microbubble injection alone (*p* < 0.05) [7]. In a preclinical rat model of HCC treated using AVUS, there was a 54% reduction in peak enhancement and perfusion index, which was significantly greater than ultrasound or microbubble controls alone [4]. In a preclinical mouse model of HCC, there was a notable decrease in peak enhancement (23.8 vs. 1.8) and perfusion index (24.3 vs. 1.1) in the AVUS group, which was not demonstrated with the injection of microbubbles alone [6].

Combining AVUS with other anticancer therapies has been demonstrated to have synergistic effects on HCC. In a preclinical rat model of HCC, there was a 170% reduction in tumor growth 7 days post-treatment and a three-fold improvement in median survival time when radiotherapy was combined with AVUS compared to radiotherapy alone [8]. Similar synergies have been seen in combining AVUS with chemotherapy. In a mouse model of HCC, combining AVUS with doxorubicin was significantly more effective at controlling tumor angiogenesis than doxorubicin or an antivascular endothelial growth factor receptor 2 (anti-VEGFR2) antibody alone [12]. In a mouse model of HCC, AVUS combined with sorafenib was significantly more effective at controlling tumor growth than AVUS or sorafenib alone (*p* < 0.01) [17]. In a rabbit model of liver cancer, combining doxorubicin with AVUS demonstrated a significant decrease in tumor size and a higher reduction in the percentage of viable tumor cells compared to AVUS or doxorubicin alone [18]. In a mouse model of HCC, combining doxorubicin with AVUS demonstrated a significant improvement in treatment efficacy compared to AVUS or doxorubicin alone, as evidenced by a higher tumor inhibition rate (80%), the lowest tumor volume growth rate (20%), and the highest survival rate [15].

The timing of AVUS relative to other therapies is likely important. Low-dose AVUS would be optimal for the neoadjuvant setting to optimize the delivery of therapeutic agents. At the same time, high-dose AVUS would be expected to be best for the adjuvant setting in which destruction of the residual tumor volume is desired. This difference was noted in a preclinical study examining the use of high-dose AVUS in combination with a percutaneous ethanol injection (PEI) in a rabbit liver cancer model [11]. PEI followed by high-dose AVUS as an adjuvant demonstrated inhibition of liver tumor growth, suppression of tumor metastases and prolonged survival [11]. The use of high-dose AVUS in the neoadjuvant setting prior to PEI resulted in reduced efficacy of PEI and worse outcomes than PEI alone, presumably related to impaired diffusion of ethanol in the tumor after AVUS. This suggests that the timing of AVUS relative to other therapies can impact treatment effectiveness.

Table S1 summarizes the evaluation of AVUS in pre-clinical studies. In summary, research has established that combining AVUS with pharmaceutical agents can achieve significantly better tumor inhibition than standalone pharmaceutical therapies [11,12,15,17,18]. Moreover, AVUS may potentiate a response to radiation therapy [8]. Initial comparisons of different dosing schemes and ultrasound intensity have been studied to elucidate the dose-dependent nature of AVUS [3,4,5,7,16]. However, there is a paucity of factor-controlled comparisons of ultrasound parameters, microbubbles, or treatment timing. As a novel treatment modality, AVUS will benefit from further study in hardware design and treatment planning.

## 8. Clinical Evidence for AVUS in Liver Tumors

**Figure 7:**
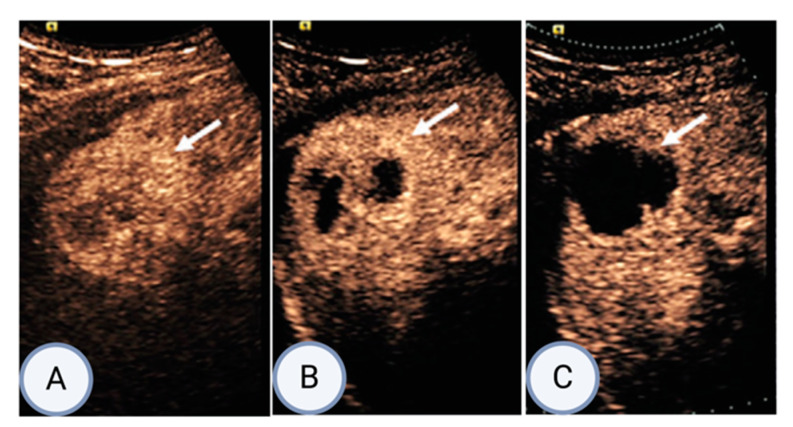
Example series adapted from Eisenbrey et al., showing post-procedure responses in a 54-year-old male participant undergoing hepatocellular carcinoma radioembolization with US-triggered microbubble (MB) destruction [19]. The series shows peak contrast-enhanced US enhancement in targeted lesion (arrow) 2 h (**A**), 1 week (**B**), and 2 weeks (**C**) after combined AVUS and radioembolization therapy.

There is limited clinical evidence for the efficacy of AVUS in treating liver tumors. In the only clinical trial of AVUS for HCC, 11 patients underwent treatment with transarterial radioembolization (TARE) alone, while 17 patients received TARE along with adjuvant AVUS immediately following TARE [19]. The reported objective response rate was significantly higher in the group that received adjuvant AVUS after TARE (93%) compared to the group treated solely with TARE (50%), (*p* = 0.02) [19]. A representative image from this study (Figure 7) demonstrates an effective reduction in perfusion and tumor volume, visualized using CEUS [19].

This clinical trial demonstrated the safety profile of AVUS in combination with TARE, with no hemodynamic or liver function test (LFT) changes observed 1 month after therapy [19]. Additionally, greater tumor responses were demonstrated in combined therapy, providing promising preliminary results [19]. Longer-term follow-up of participants is needed to further validate the safety of AVUS in HCC patients, and larger prospective trials are needed to establish best clinical practice in using novel AVUS in the context of HCC.

## 9. Current Status and the Limiting Factors of AVUS

AVUS is a promising emerging therapeutic technology that has seen significant progress in recent years in treating HCC. Studies from different groups have demonstrated its effectiveness. The current therapeutic systems use standalone therapeutic transducers and electronics, which are often not easily accessible to users and are difficult to use in a clinical setting. Efforts are underway to integrate therapeutic treatment and noninvasive monitoring imaging. However, its development is still in its early stages, and its long-term potential will depend on its ability to demonstrate improved patient outcomes and drive advancements in the standard of care.

The efficacy of AVUS depends on the degree of tumor vascularity and its susceptibility to damage by the AVUS treatment parameters. Tumors with faster blood flow might exhibit a weaker response to treatment due to the reduced interaction between microbubbles and ultrasound caused by the rapid transit of the microbubbles through the ultrasound beam [5]. In addition, the heterogeneity in the vascular structure of tumors with vascular shunting could pose a challenge to the response of AVUS therapy if the microbubbles have insufficient dwell time in the ultrasound treatment area. Tumors often exhibit a mix of “early immature” and “late mature” vessels, and this composition varies across tumors and their developmental stages. Consequently, the response of tumors to AVUS might differ based on the specific architecture of their vascular network and the tumor stage of development [3]. However, the real-time assessment of treatment efficacy provides opportunities for adjusting the treatment to maximize the effects on the tumor vasculature. For example, dose parameters and treatment fields can be adjusted to first thrombose an intratumoral vascular shunt and then adjusted to allow for increased permeability and drug delivery. Although CEUS has been demonstrated to be a safe technique due to the low likelihood of adverse reactions to microbubbles such as SonoVue^®^, further studies are needed to establish contraindications specific to AVUS [20]. Similar to CEUS, a known history of allergic reactions to microbubbles, severe pulmonary hypertension, and pregnancy may limit the application of AVUS [20]. Finally, the technique may not be feasible in patients with a large body habitus, consequently decreasing visibility and limited ultrasound treatment windows.

## 10. Conclusions

Antivascular ultrasound (AVUS) is a promising new noninvasive approach in the treatment of hepatocellular carcinoma (HCC), which can be used alone or as a neoadjuvant or adjuvant therapy in combination with other treatments. By combining low-intensity ultrasound and microbubbles, AVUS disrupts tumor vascularity. Its selective effects on tumor vasculature and potential for improving drug delivery positions AVUS as a valuable therapeutic modality in treating HCC. Although we have presented a dozen pre-clinical studies demonstrating the potential of AVUS in modulating tumor perfusion, study designs vary considerably. Moreover, long-term follow-up of existing clinical trial patients and more prospective clinical studies are needed to further establish the safety profile of AVUS and explore the potential of this promising noninvasive procedure in the treatment of HCC.

## Data Availability

Data are contained within the article.

## Supplementary Materials

The following supporting information can be downloaded at: https://www.mdpi.com/article/10.3390/cancers16223756/s1, Table S1: Summary of pre-clinical and clinical evidence reviewed pertaining to the use of AVUS in HCC.
